# Effect of an Aerosol Box on Intubation in Simulated Emergency Department Airways: A Randomized Crossover Study

**DOI:** 10.5811/westjem.2020.8.48901

**Published:** 2020-09-24

**Authors:** Joseph S. Turner, Lauren E. Falvo, Rami A. Ahmed, Timothy J. Ellender, Dan Corson-Knowles, Anna M. Bona, Elisa J. Sarmiento, Dylan D. Cooper

**Affiliations:** Indiana University School of Medicine, Department of Emergency Medicine, Indianapolis, Indiana

## Abstract

**Introduction:**

The use of transparent plastic aerosol boxes as protective barriers during endotracheal intubation has been advocated during the severe acute respiratory syndrome coronavirus 2 pandemic. There is evidence of worldwide distribution of such devices, but some experts have warned of possible negative impacts of their use. The objective of this study was to measure the effect of an aerosol box on intubation performance across a variety of simulated difficult airway scenarios in the emergency department.

**Methods:**

This was a randomized, crossover design study. Participants were randomized to intubate one of five airway scenarios with and without an aerosol box in place, with randomization of intubation sequence. The primary outcome was time to intubation. Secondary outcomes included number of intubation attempts, Cormack-Lehane view, percent of glottic opening, and resident physician perception of intubation difficulty.

**Results:**

Forty-eight residents performed 96 intubations. Time to intubation was significantly longer with box use than without (mean 17 seconds [range 6–68 seconds] vs mean 10 seconds [range 5–40 seconds], p <0.001). Participants perceived intubation as being significantly more difficult with the aerosol box. There were no significant differences in the number of attempts or quality of view obtained.

**Conclusion:**

Use of an aerosol box during difficult endotracheal intubation increases the time to intubation and perceived difficulty across a range of simulated ED patients.

## INTRODUCTION

There have been numerous recommendations for enhanced personal protective equipment (PPE) during endotracheal intubation during the severe acute respiratory syndrome coronavirus 2 (SARS-CoV-2) pandemic.^1^–^3^ Transparent “aerosol boxes” have been promoted as additional barriers to prevent droplet spread during endotracheal intubation.^4^–^6^ Decreased spread of simulated droplet particles has been demonstrated with the use of such a box during a cough simulation.^4^ Although aerosol boxes have received extensive attention on social media and there is evidence of worldwide distribution of such devices,^7^ some have cautioned against widespread implementation without further research into potential negative effects.^8^

Initial proponents have since noted restricted movement with aerosol boxes.^4^,^6^ Begley et al conducted a simulation study in which they demonstrated an increased time to intubation with boxes.^7^ To date, most of the studies regarding these extended protection measures have been conducted in simulated operating room or intensive care unit settings and have focused on conventional airways. The need for reliable protection for physicians is particularly urgent in the chaotic frontline of the emergency department (ED), where the frequency of difficult intubations and the undifferentiated patients could amplify both the downsides and benefits of aerosol boxes. The objective of this study was to measure the effect of an aerosol box on intubation performance across a variety of simulated difficult airway scenarios in the ED.

## METHODS

### Study Design and Population

This was a randomized, crossover design study conducted at a large, university-affiliated simulation center. Study participants included resident physicians from all years of a three-year emergency medicine (EM) program (with the additional inclusion of participants from a five-year combined EM-pediatrics program). Each participant signed an informed consent statement. The study was deemed exempt by the university’s institutional review board.

### Study Protocol and Materials

Faculty instructors from our department’s Division of Simulation developed five patient case scenarios using Laerdal SimMan 3G (Laerdal Medical, Stavanger, Norway) to simulate one normal airway and four difficult airways based on real-life patients seen during the SARS-CoV-2 pandemic. These included the following: 1) an angioedema patient simulated using the large tongue function on the mannequin; 2) a morbidly obese patient simulated by adding pillows, ACE wrap, and skin-colored padding to the torso and neck of the mannequin (which partially limited neck mobility and also caused the mannequin’s neck to be slightly flexed while in the supine position); 3) a trauma patient simulated with the mannequin on a backboard and wearing a cervical collar; and 4) an upper gastrointestinal (GI) bleed patient using a modified Laerdal SimMan that has been previously described.^9^

Participating residents were divided into 21 small groups of 2–4 residents based on assignments for a concurrent procedure lab that was part of their standard curriculum. Each study group was randomized by an electronic number generator to one of the five patient types. Each resident performed two intubations on their patient type, with sequence of control vs intervention randomized by an electronic number generator. Intubation with the aerosol box in place served as the intervention; intubation without a box was the control. Our aerosol box was a 20” × 20” × 16” Plexiglass structure with 4”-diameter arm holes, approximately nine kilograms, manufactured at our institution and based on the original design from Taiwan^10^ that was studied by Canelli et al.^4^

**Population Health Research Capsule**

What do we already know about this issue?

*Aerosol boxes may decrease droplet spread of coronavirus but may increase intubation time in controlled settings. Effects in emergency airways are unknown*.

What was the research question?

Does use of an aerosol box interfere with emergency endotracheal intubation in simulated undifferentiated difficult airways?

What was the major finding of the study?

*Aerosol box usage increased perceived difficulty and time to intubation for simulated difficult emergency intubations*.

How does this improve population health?

*Quantifying the increased difficulty of emergency intubation with intubation boxes will inform development of airway protocols for infection control during pandemics*.

A concurrent media access control (C-MAC) video laryngoscope was used for all intubations (Karl Storz SE & Co., Tuttlingen, Germany) since this is the standard practice for all potential SARS-CoV-2 intubations at our institution. Size 3 and size 4 standard curved blades and a hyper-angulated blade were available. Endotracheal tubes (ETT) with both flexible and rigid stylets were provided. A gum-elastic bougie was available to all upper level residents; interns were not provided this device given their lack of previous training with it. To increase resident familiarity with the box, participants practiced intubating a normal 3G mannequin through the aerosol box with both a normal curved blade and a hyper-angulated blade for five minutes. For subsequent data collection, participants intubated their randomly assigned patient type in video-recorded attempts both with and without the box and using any of the available equipment.

### Data Collection and Outcomes

The primary outcome was time to intubation. For all recorded attempts, a faculty investigator timed the intubation on site, from the time the resident picked up the blade until the ETT passed through the vocal cords per a previously published protocol.^11^ Faculty recorded this time in seconds as well as number of attempts (defined as number of times the blade was placed into the patient’s mouth). Residents recorded Cormack-Lehane (CL) view, percent of glottic opening (POGO) score, and their perceived difficulty of intubation on a 10-point Likert scale. They also provided open-ended comments about the intubation immediately after the attempt. See Appendix A for the complete data-collection instrument.

Time to intubation, CL view, and POGO score were independently reviewed by one of the faculty investigators not involved in initial data collection, using recorded video of the C-MAC screen. Discrepancies from the original recorded data were reviewed and discussed by the entire study group until consensus was obtained.

### Statistical Analysis

We summarized frequencies and percentages by group for categorical variables. Continuous variables were summarized by group using median and range. We used chi-square test, Fisher’s exact test, and Wilcoxon test to test for differences between groups. We performed all statistical analysis using SAS Version 9.4 (SAS Institute, Cary, NC).

## RESULTS

Forty-eight residents performed 96 intubations (Table 1). Time to intubation was significantly longer with the aerosol box in the full cohort of patients, as well as with the trauma, obese, and angioedema patient subgroups (Table 2). The point estimate for time to intubation was also longer with the box in the normal patient and GI bleed patients but did not reach statistical significance. Only two intubations required multiple attempts, both with box use. Participants rated intubation with the box as being significantly more difficult. There was no statistically significant difference between groups for number of attempts, CL view, or POGO score.

Participants volunteered comments on 58 intubations (40 intubations with the box, 18 without). One of the study investigators (JT) categorized comments according to themes. Major themes with representative example comments are displayed in Table 3. The most common comments involved restricted movement or difficulty with equipment when using the box. Thirteen responses mentioned decreased space or maneuverability in the box, while seven additional comments specifically noted equipment issues when using the box (such as cord tangle or ETT contact with the box). Three comments indicated that using the box was easier than the participant anticipated. There were no comments pertaining to the view obtained.

## DISCUSSION

Time to intubation was longer with aerosol box use in our simulated difficult airway scenarios. We chose time to intubation as our primary endpoint because rates of hypoxia are high during intubation of patients with SARS-CoV-2,^12^ increasing the importance of limiting apneic time in this patient population. Similar to Begley et al,^7^ our study demonstrated a significantly increased time to intubation with the use of an aerosol box.

We sought to test aerosol boxes across a variety of airway types commonly encountered in the ED. It is possible that the magnitude of disadvantage from box use is greater in some patient types than others, altering the risk-benefit assessment. Accordingly, we randomized the type of patient that participants would intubate. Participants also had equipment that replicated current use in our ED, to include a video laryngoscope with normal and hyper-angulated blades. These elements more realistically simulated the variability of ED practice than previous aerosol-box studies.

Protecting physicians during intubations is critical in the time of the SARS-CoV-2 pandemic. Aerosol boxes may offer some protection by reducing pathogen spread.^4^ Initiatives to quickly develop protective equipment, aided by social media and 3-D printing technology, have delivered multiple versions of aerosol boxes to hospitals across the country. However, the advantages of box use must be balanced against their negative impacts. In addition to longer intubation times, participants in our study rated intubation as more difficult with the box. The increased perceived difficulty correlated with the main concern voiced by participants, that of difficulty maneuvering equipment within the box. This is consistent with reports from other studies.^4^,^6^ Our study was not powered to detect difference in first-pass success, but both intubations that required multiple attempts in our study involved box use. This is also consistent with the findings of Begley et al.^7^

It is possible that these issues could be mitigated by improved box design or additional practice. Several participants in our study noted that intubation with the box became easier with practice. Future studies could better define the amount of training required with aerosol boxes to develop provider proficiency. Until that time, consistent with the recommendation of other investigators,^7^,^8^ we caution against widespread adoption of these devices.

## LIMITATIONS

This study was conducted at a single institution with EM residents trained at a single residency program. While participants had a broad range of airway experience from relatively novice interns to upper-level residents with more than 100 intubations, it is not ear whether clinicians with additional experience, including attending physicians, would be similarly affected by use of the box. Although there was a significant difference in the primary outcome even in our most experienced intubators, the magnitude of this difference was smaller than with our less experienced participants (Appendix B). Additionally, only one brand of video laryngoscope was used in the assessment, and intubations were in a simulated setting. These factors may also limit generalizability. We used an older box design, and it is possible that newer designs may result in better performance than the older design.^7^,^13^,^14^

To limit confounding variables, residents did not have to move the box on and off the bed in our study, which could affect time to the intubation. In addition, we used a custom perception-of-difficulty scale that has not been validated in external studies. It was not possible to blind the residents to the intervention and data collection, so resident preconceived biases may have affected their performance. Finally, as with all simulation airway studies, the movement used to intubate mannequins does not exactly replicate the movement used in human patients. It is, therefore, possible that the effects of the box would be different in the emergency department compared to the simulation laboratory.

## CONCLUSION

Use of an aerosol box during difficult endotracheal intubation increases the time to intubation across a range of simulated ED patients.

## Supplementary Information





## Figures and Tables

**Table 1:** Study characteristics (N = 96) of residents and the simulated intubations they performed with and without a transparent aerosol box.

	No box used	Box used	P-value*
Postgraduate year			1.00
1	21 (43.7)	21 (43.7)	
2	5 (10.4)	5 (10.4)	
3–5	22 (45.8)	22 (45.8)	
Blade used			0.6820
Normal	23 (47.9)	21 (43.7)	
Hyper-angulated	25 (52.1)	27 (56.2)	
Patient type			1.00
Normal	11 (22.9)	11(22.9)	
Trauma/cervical collar	10 (20.8)	10 (20.8)	
Obese	10 (20.8)	10 (20.8)	
Angioedema	10 (20.8)	10 (20.8)	
Gastrointestinal bleed	7 (14.6)	7 (14.6)	
Bougie			0.6170
No	47 (97.9)	45 (93.7)	
Yes	1 (2.1)	3 (6.2)	

* Estimated using chi-square or Fisher’s exact test.

**Table 2:** Time to intubation results, median (minimum-maximum).

	No box used	Box used	P-value
Time (in seconds)	10 (5.0–40.0)	17.0 (6.0–68.0)	<.0001
Normal	10.0 (6.0–23.0)	12.0 (9.0–68.0)	0.0746
Trauma/cervical collar	7.0 (5.0–40.0)	11.0 (7.0–23.0)	0.0272
Obese	10.0 (7.0–29.0)	18.5 (12.0–29.0)	0.0079
Angioedema	9.5 (7.0–18.0)	21.5 (6.0–66.0)	0.0113
Gastrointestinal bleed	15.0 (12.0–21.0)	18.0 (14.0–25.0)	0.1391
Number attempts	1.0 (1.0–1.0)	1.0 (1.0–2.0)	0.1595
Difficulty	3.0 (1.0–7.0)	4.0 (1.0–9.0)	0.0008
Cormack-Lehane view	1.0 (1.0–2.0)	1.0 (1.0–2.0)	0.4154
Percent of glottic opening	100.0 (50.0–100.00)	95.0 (20.0–100.0)	0.1576

* Estimated using Wilcoxon test.

**Table 3:** Open-ended comments regarding use of transparent aerosol box during intubations.

Theme	Decreased space/maneuverability	Equipment issues	Easier than anticipated
Representative comments	“Hand motions more difficult and limited due to box”	“Got cord tangled once blade was in box; had to remove blade and restart”	“Somewhat limiting but easy to navigate with a few practice attempts”
	“Box was difficult to maneuver in”	“Cord length with C-MAC is a problem depending on which box hole you thread blade through”	“Still relatively easy”

## References

[1] Cheung JC, Ho LT, Cheng JV (2020). Staff safety during emergency airway management for COVID-19 in Hong Kong. Lancet Respir Med.

[2] Cook TM, El-Boghdadly K, McGuire B (2020). Consensus guidelines for managing the airway in patients with COVID-19: Guidelines from the Difficult Airway Society, the Association of Anaesthetists the Intensive Care Society, the Faculty of Intensive Care Medicine and the Royal College of Anaesthetists. Anaesthesia.

[3] Orser BA (2020). Recommendations for endotracheal intubation of COVID-19 patients. Anesth Analg.

[4] Canelli R, Connor CW, Gonzalez M (2020). Barrier enclosure during endotracheal intubation. N Engl J Med.

[5] Lai YY, Chang CM (2020). A carton-made protective shield for suspicious/confirmed COVID-19 intubation and extubation during surgery. Anesth Analg.

[6] Leyva Moraga FA, Leyva Moraga E, Levya Moraga F (2020). Aerosol box, an operating room security measure in COVID-19 pandemic. World J Surg.

[7] Begley J, Lavery K, Nickson C (2020). The aerosol box for intubation in COVID-19 patients: an in-situ simulation crossover study. Anaesthesia.

[8] Kearsley R (2020). Intubation boxes for managing the airway in patients with COVID-19. Anaesthesia.

[9] Cooper D, Avera B, Hasty G (2015). Difficult airway: simulating a massive GI bleed. Simul Healthc.

[10] Everington K (2020). Taiwanese doctor invents device to protect US doctors against coronavirus.

[11] Turner JS, Ellender TJ, Okonkwo ER (2017). Cross-over study of novice intubators performing endotracheal intubation in an upright versus supine position. Intern Emerg Med.

[12] Yao W, Wang T, Jiang B (2020). Emergency tracheal intubation in 202 patients with COVID-19 in Wuhan, China: lessons learnt and international expert recommendations. Br J Anaesth.

[13] Brown S, Patrao F, Verma S (2020). Barrier system for airway management of COVID-19 patients. Anesth Analg.

[14] Malik JS, Jenner C, Ward PA (2020). Maximising application of the aerosol box in protecting healthcare workers during the COVID-19 pandemic. Anaesthesia.

