# Supplementary material for: Effect of an Aerosol Box on Intubation in Simulated Emergency Department Airways: A Randomized Crossover Study
Source: West J Emerg Med. 2020 Sep 24;21(6):78–82. doi: 10.5811/westjem.2020.8.48901 (PMC7673888; doi:10.5811/westjem.2020.8.48901)
Supplement: Supplementary file 1 [file wjem-21-78-s001.docx]

**Appendix A**

Last 4 Digits Phone # __________

**INTUBATION #1**

1. Group: A B
2. Circle your level of training:

PGY-1 PGY-2 PGY-3-5

1. Please circle: Box Used Box Not Used
2. Please circle any that you used:

Video scope (normal mac blade) Video scope (hyper-angulated blade)

Bougie Fiberoptic scope

1. Was intubation successful: Y N
2. Number of attempts (filled out by faculty preceptor): __________
3. Total time to intubation (filled out by faculty preceptor): __________
4. Case type (filled out by faculty preceptor):

Normal Obese C-collar GI-Bleed Large Tongue

1. Please circle your perceived level of difficulty of the intubation you performed:

(Very 1 2 3 4 5 6 7 8 9 10 (Very

Easy) (Moderately Difficulty) Difficult)

1. Please circle the view that most closely resembles the best view you were able to obtain during the procedure:


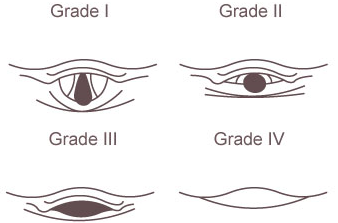


Grade 1 = Complete glottis visible

Grade 2 = Partial glottis visible (anterior not seen)

Grade 3 = Epiglottis seen, but not glottis

Grade 4 = Epiglottis not seen

1. Estimate the percentage of the glottis (see above) that you could visualize: ________ (0-100%)
2. Please provide any additional comments about this intubation experience:

Last 4 Digits Phone # __________

**INTUBATION #2**

1. Group: A B
2. Circle your level of training:

PGY-1 PGY-2 PGY-3-5

1. Please circle: Box Used Box Not Used
2. Please circle any that you used:

Video scope (normal mac blade) Video scope (hyper-angulated blade)

Bougie Fiberoptic scope

1. Was intubation successful: Y N
2. Number of attempts (filled out by faculty preceptor): __________
3. Total time to intubation (filled out by faculty preceptor): __________
4. Case type (filled out by faculty preceptor):

Normal Obese C-collar GI-Bleed Large Tongue

1. Please circle your perceived level of difficulty of the intubation you performed:

(Very 1 2 3 4 5 6 7 8 9 10 (Very

Easy) (Moderately Difficulty) Difficult)

1. Please circle the view that most closely resembles the best view you were able to obtain during the procedure:


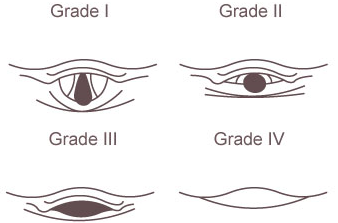


Grade 1 = Complete glottis visible

Grade 2 = Partial glottis visible (anterior not seen)

Grade 3 = Epiglottis seen, but not glottis

Grade 4 = Epiglottis not seen

1. Estimate the percentage of the glottis (see above) that you could visualize: ________ (0-100%)
2. Please provide any additional comments about this intubation experience:
