# Supplementary material for: Effect of an Aerosol Box on Intubation in Simulated Emergency Department Airways: A Randomized Crossover Study
Source: West J Emerg Med. 2020 Sep 24;21(6):78–82. doi: 10.5811/westjem.2020.8.48901 (PMC7673888; doi:10.5811/westjem.2020.8.48901)
Supplement: Supplementary file 2 [file wjem-21-78-s002.docx]

| **Appendix B.** Time differences by PGY, Median (Min-Max). | | | |
| --- | --- | --- | --- |
|  | **No Box Used** | **Box Used** | **P-value*** |
| PGY 1 | 12.0 (7.0-40.0) | 21.0 (12.0-66.0) | **0.0002** |
| PGY 2 | 10.0 (6.0-13.0) | 14 (10.0-68.0) | 0.0562 |
| PGY 3 | 9.0 (5.0-21.0) | 12.5 (6.0-25.0) | **0.0284** |
| *Estimated using Wilcoxon test | | | |

PGY = post-graduate year
